# P-1614. Peak SARS-CoV-2 and influenza A Viral Loads Relative to Symptom Onset, 2023-2025: Impact of Vaccination and Implications for Multiplexed Testing

**DOI:** 10.1093/ofid/ofaf695.1792

**Published:** 2026-01-11

**Authors:** Joanne Hunt, Gregory L Damhorst, Jennifer Frediani, Richard B Parsons, Adrianna Westbrook, Kaleb McLendon, Wilbur A Lam, Julie Sullivan, Greg S Martin, Nira Pollock

**Affiliations:** Rollins School of Public Health, Atlanta, Georgia; Emory University, Atlanta, GA; Emory University, Atlanta, GA; Emory University, Atlanta, GA; Emory, Atlanta, Georgia; Emory University, Atlanta, GA; Emory University School of Medicine/Georgia Institute of Technology, Atlanta, GA; Emory University, Atlanta, GA; Emory University School of Medicine, Atlanta, Georgia; Boston Children's Hospital, Boston, MA

## Abstract

**Background:**

We previously reported that nasal SARS-CoV-2 viral load peaked around the fourth day of symptoms in a highly immune population sampled April 2022 – April 2023, while influenza A viral loads peaked soon after symptom onset. We hypothesized that SARS-CoV-2 kinetics may have changed in subsequent years due to reduced incidence of infections and varying vaccination patterns. We also reexamined viral kinetics in influenza A infections.

**Figure 1.**
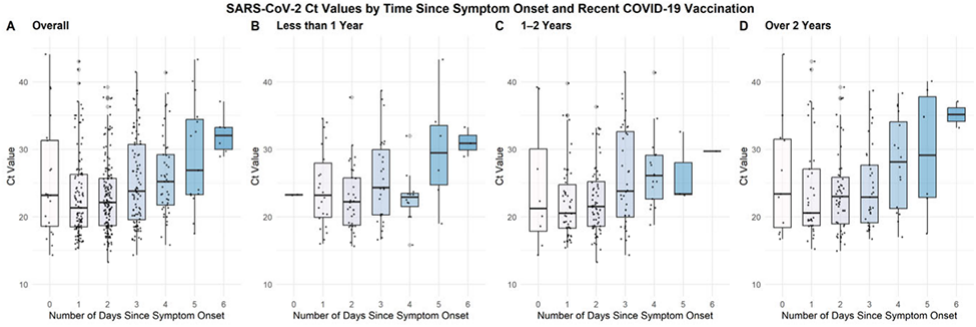
SARS-CoV-2 Ct Values by Time Since Symptom Onset and Recent COVID-19 Vaccination SARS-CoV-2 Ct values measured in nasal swab samples plotted by days since symptom onset for PCR-positive symptomatic adults (day 0 = the first day of symptoms). A, full cohort. B-D, Subgroups categorized by time since most recent COVID-19 vaccination. Ct, Cycle threshold.

**Figure 2.**
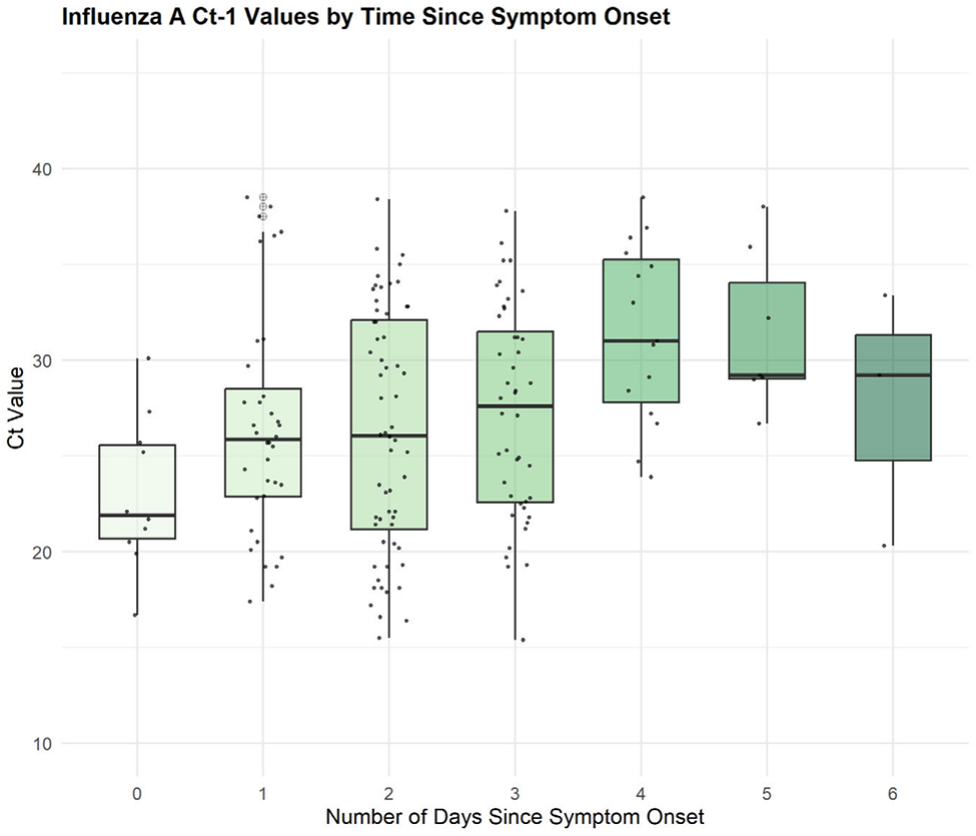
Influenza A Ct-1 Values by Time Since Symptom Onset Influenza A Ct values measured in nasal swab samples plotted by days since symptom onset for PCR-positive symptomatic adults (day 0 = the first day of symptoms). Ct, cycle threshold.

**Methods:**

Participants with symptomatic upper respiratory infection were recruited at hospital and community-based testing centers in Georgia between April 14, 2023 – April 13, 2025. Participants reported date of symptom onset and vaccination history. A nasal swab specimen collected by a healthcare worker was tested on the Xpert® Xpress CoV-2/Flu/RSV plus assay (GeneXpert system); Ct values were recorded. Analysis was limited to participants ages 16 and older who tested positive for SARS-CoV-2 or influenza A.

**Results:**

581 eligible participants tested positive for SARS-CoV-2 and Ct values were available for 579 (99.7%). SARS-CoV-2 vaccination information was available for 441 participants, of whom 106 (24.0%) had most recently received vaccination within one year, 179 (40.6%) 1-2 years prior, and 156 (35.8%) > 2 years prior. In the full cohort, lowest median SARS-CoV-2 Ct values (reflecting highest viral load) were observed on the 2^nd^ day of symptoms, but in subgroup analysis lowest Ct values were observed on the 3^rd^ day in those vaccinated within the last year and the 2^nd^ day in those vaccinated more than 1 year prior (Fig. 1). 187 participants tested positive for influenza A (181 [97%] with Ct values). The lowest median influenza A Ct values were observed on the 1st day of symptoms (Fig. 2).

**Conclusion:**

In the full cohort tested April 2023-2025, median SARS-CoV-2 viral loads appeared to peak earlier compared to a cohort tested the preceding year. Notably, the timing of last vaccination appeared to impact timing of peak viral load, again suggesting that more robust immunity correlates with earlier symptom onset relative to peak. Again, median influenza A viral loads peaked on the first day of symptoms. This information may inform strategies to optimize use of over-the-counter multiplexed antigen tests.

**Disclosures:**

Wilbur A. Lam, MD, PhD, Sanguina, Inc: Board Member|Sanguina, Inc: several patents that have come from my lab|Sanguina, Inc: Stocks/Bonds (Private Company)

